# Prognostic value of long non-coding RNA GHET1 in cancers: a systematic review and meta-analysis

**DOI:** 10.1186/s12935-020-01189-9

**Published:** 2020-04-07

**Authors:** Dingding Wang, Hong Zhang, Xiaolian Fang, Xue Zhang, Honggang Liu

**Affiliations:** 1 Department of Pathology, Beijing Tongren Hospital, Capital Medical University, Beijing, 100730 China; 2 Department of Otolaryngology, Head and Neck Surgery, Beijing Children’s Hospital, Capital Medical University, National Center for Children’s Health, No.56 Nanlishi Rd, Beijing, 100045 China

**Keywords:** GHET1, Meta-analysis, Cancers, Prognosis

## Abstract

**Background:**

A number of studies have demonstrated the critical role of long non-coding RNA gastric cancer high expressed transcript 1 (GHET1) in many cancers. This meta-analysis provides an evidence-based evaluation of the prognostic role of GHET1 in cancer.

**Materials and methods:**

Literature searches were conducted in several databases including Medline, Cochrane, EMBASE, CNKI, and Wanfang. The pooled odds ratio (OR) and hazard ratio (HR) with 95% confidence interval (CI) were used to evaluate the role of GHET1 in cancer. The study protocol was registered at PROSPERO (ID: CRD42018111252).

**Results:**

Sixteen studies, containing 1315 patients, were analyzed in this meta-analysis. The pooled results indicated that GHET1 overexpression was significantly associated with poor overall survival (OS) and disease-free survival (DFS) in cancer. Moreover, up-regulation of GHET1 expression predicted larger tumor size, positive lymph node metastasis, positive distant metastasis, and advanced TNM (tumor-node-metastases) stage in human cancers.

**Conclusion:**

There is a significant correlation between up-regulation of GHET1 and both poor prognosis and advanced clinicopathological cancer characteristics. GHET1 may be a potential prognostic predictor for human cancers.

## Background

Despite great progress in cancer diagnosis and treatment, there were still 18.1 million new cancer cases and 9.6 million cancer deaths worldwide in 2018 [1]. The treatment prognosis of most cancers remains poor; one of the primary reasons for this is the lack of specific biomarkers for the early diagnosis of most cancers [2]. Therefore, it is necessary to identify novel prognostic markers of cancer for potential clinical application [3–5].

Recently, many studies have indicated that long non-coding RNAs (lncRNAs) play a crucial role in the progression of cancer [6–10]. Some lncRNAs are involved in the modulation of cancer proliferation, invasion, and metastasis. In addition, several studies have found that lncRNAs are potential cancer-specific prognostic biomarkers [2, 11–13]. The lncRNA gastric cancer high expressed transcript 1 (GHET1) is located on chromosome 7q36.1 and was originally found to be highly expressed in gastric cancer [14]. In gastric cancer, the up-regulation of GHET1 promotes tumor cell proliferation in vitro and in vivo by physically binding to IGF2BP1, thereby enhancing the interaction between c-Myc mRNA and IGF2BP1; this can enhance the stability of c-Myc mRNA [14].

Clinically, several studies have indicated that up-regulation of GHET1 is associated with poor prognosis and advanced clinical features in several cancers [14–24]. Most existing studies suggest that GHET1 might be a potential biomarker for predicting the prognosis of human cancers. However, due to limitations such as small sample sizes and discrete outcomes, the findings of a single study may not accurately capture the phenomenon under examination [14–24]. Thus, we undertook a systematic review and meta-analysis of all eligible studies to perform an evidence-based evaluation of the prognostic role of GHET1 in cancer.

## Materials and methods

This systematic meta-analysis was conducted accordance with the Preferred Reporting Items for Systematic Reviews and Meta-Analyses (PRISMA) guidelines. It has been registered with PROSPERO (ID: CRD42018111252).

## Literature search and selection

Literature searches were conducted in several databases, including Medline, Cochrane, EMBASE, CNKI, and Wanfang, up until April 15th, 2019. The search strategy was as follows: “GHET1” OR “lncRNA GHET1“ OR “gastric cancer high expressed transcript 1“) AND (“cancer“ OR “neoplasm“ OR “tumor“ OR “carcinoma“). The search was limited to English and Chinese studies. The references of relevant studies were also retrieved to avoid missing any potentially eligible studies.

## Inclusion and exclusion criteria

The inclusion criteria for this meta-analysis were as follows: (1) detection of GHET1 expression in human cancers by quantitative real-time PCR (qRT-PCR); (2) patients in the study were divided into subgroups based on different GHET1 expression levels; (3) prognosis or clinicopathological feature of GHET1 was reported; (4) hazard ratios (HRs) and 95% confidence intervals (CI) were able to be obtained directly or indirectly from the article. In addition, the exclusion criteria were as follows: (1) reviews, editorials, conference reports, case reports, and meta-analyses; (2) non-human tissue studies; (3) studies only investigating the molecular mechanisms of GHET1; (4) duplicate publications. The titles and abstracts were first evaluated based on the inclusion and exclusion criteria; the full texts of those reports that appeared to meet the criteria were then further evaluated.

## Data extraction and quality assessment

Two researchers (Dingding Wang and Xiaolian Fang) independently extracted data from the selected studies according to uniform data extraction standards; any disagreements were settled by consensus with a third investigator (Hong Zhang). Extracted data included: first author’s name, publication year, country or region, sample size, cancer type, method for detection of GHET1, cut-off values, treatment data, disease-free survival (DFS), overall survival (OS), and clinical stage of cancer. If the HRs and 95% CIs for DFS or OS were not available in the paper, the data were indirectly extracted from survival curves, based on the approach described previously [25]. The Newcastle–Ottawa Scale (NOS) was used to assess the methodological quality of each study. A study was considered to be high quality if the NOS score was greater than or equal to 6; otherwise, it was considered to be a low-quality study.

## Public data and tools

TCGA data, including RNAseqV2 and clinical data, were extracted from the TCGA Data Portal and UCSC Xena project, according to the publication guidelines (http://cancergenome.nih.gov/publications/publicationguidelines). GEPIA was used to analyze RNAseq data. Differential expression analysis was conducted using one-way ANOVA. Survival analysis was performed using the Kaplan–Meier method and log-rank test.

## Statistical analysis

Pooled HRs and 95% CIs were used to assess the relationship between GHET1 expression and prognosis. Odd ratios and 95% CIs were combined to evaluate the relationships between GHET1 expression and clinicopathological factors. If the 95% CI of the combined OR or HR did not overlap 1, the result was considered statistically significant. The I^2^ and Q tests were used to evaluate the heterogeneity of the meta-analysis. If I^2^ > 50% or P < 0.05, heterogeneity was considered statistically significant and a random effects model was chosen; otherwise, a fixed effects model was utilized. We also performed a sensitivity analysis to evaluate the stability of the combined results. The Begg’s test was used to assess potential publication bias. All statistical calculations were performed using RevMan 5.3 software and STATA software version 14.2 (StataCorp LLC, College Station, TX, USA). Moreover, Engauge Digitizer 10.0 was utilized to extract HRs and 95% CIs from the Kaplan–Meier survival curves. A P value of less than 0.05 was considered to be statistically significant.

## Results

### Study characteristics

**Fig. 1:**
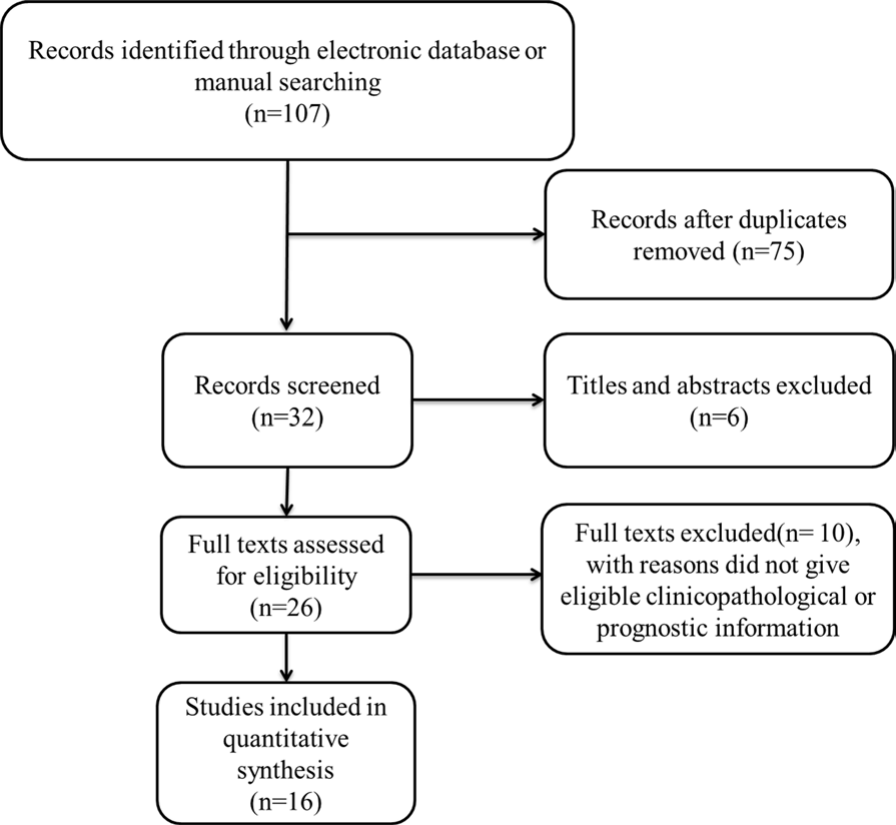
Flaw chart of literature search and selection

A preliminary literature search was conducted based on the established search strategy; a total of 107 articles were obtained. After removing 75 duplicates, the titles and abstracts of the remaining 32 articles were evaluated according to the inclusion criteria. Following this, six studies were excluded. The full texts of the remaining 26 articles were reviewed; this resulted in the exclusion of 10 papers due to a lack of eligible prognostic or clinicopathological information. Finally, a total of 16 studies were included in the meta-analysis (Fig. 1).

The basic characteristics of the patients in these 16 studies were extracted and are summarized in Table 1. A total of 1315 patients were included in the current meta-analysis; all patients were from China. The sample size of each individual study ranged from 42 to 182, and the publication years ranged from 2014 to 2019. Fourteen of the articles were published in English and two were published in Chinese. GHET1 expression in all studies was assessed using qRT-PCR. Moreover, 13 studies provided cut-off definitions for high or low GHET1 expression groups, including the median and median ratio. However, the remaining three studies did not provide explicit cut-off values. The types of cancer evaluated in the evaluated studies included: bladder cancer, breast cancer, cervical cancer, esophageal squamous cell carcinoma, gastric cancer, hepatocellular carcinoma, non-small cell lung cancer, osteosarcoma, pancreatic cancer, and renal cell carcinoma. Patients in 14 cohorts underwent surgical treatments; however, the therapeutic approach adopted in two of the studies was not available. Meanwhile, 15 studies reported the clinical stage of the patients, 12 studies reported OS, and four studies reported DFS. The NOS score ranged from 7 to 9, with an average score of 7.7; this indicates that each of the studies employed high-quality methodology.

**Table 1:** Basic characteristics of the included studies

Author	Year	Country	Sample size (n)	Cancer type	Cut-off value	Treatments	Outcomes	HR statistic	NOS
F. Yang	2014	China	42	GC	Median	Surgery	CP, OS	Survival curve	7
H. Liu	2018	China	86	ESCC	Median	Surgery	CP, OS	Survival curve	8
H.F. Liu	2017	China	55	ESCC	Median	Surgery	CP	NA	8
H.Y. Zhou	2017	China	64	PADC	NA	Surgery	CP	NA	8
J. Li	2017	China	179	HCC	NA	Surgery	CP, DFS, OS	Survival curve	8
L. Jin	2017	China	68	HCC	NA	Surgery	CP, OS	Survival curve	7
L.J. Li	2014	China	80	BLC	Median	Surgery	OS	Survival curve	7
Q.C. Zhang	2019	China	94	cervical cancer	Median	NA	CP, OS	Data in paper	7
Q.M. Shen	2018	China	105	NSCLC	Median	NA	CP, DFS, OS	Survival curve	7
R. Song	2018	China	60	BC	Median	Surgery	CP, OS	Survival curve	8
W. Yang	2018	China	60	osteosarcoma	Median	Surgery	CP, OS	Survival curve	7
W.J. Xie	2019	China	40	RCC	Median ratio	Surgery	CP	NA	8
Y. Xia	2016	China	42	GC	Median	Surgery	CP	NA	8
Y.P. Zhang1	2018	China	106	HCC	Median	Surgery	CP, DFS, OS	Survival curve	8
Y.P. Zhang2	2018	China	182	HCC	Median	Surgery	CP, DFS, OS	Data in paper	9
Z.B. Guan	2018	China	52	NSCLC	Median	Surgery	CP, OS	Data in paper	8

*BC* breast cancer, *BLC* bladder cancer, *ESCC* esophageal squamous cell carcinoma, *GC* gastric cancer, *HCC* hepatocellular carcinoma, *PADC* pancreatic cancer, *NSCLC* non-small lung cancer, *qRT-PCR* quantitative reverse transcription polymerase chain reaction, *CP* clinicopathological parameters, *OS* overall survival, *DFS* disease-free survival, *NOS* Newcastle–Ottawa Scale, *NA* not available

### Association between GHET1 expression and prognosis

**Fig. 2:**
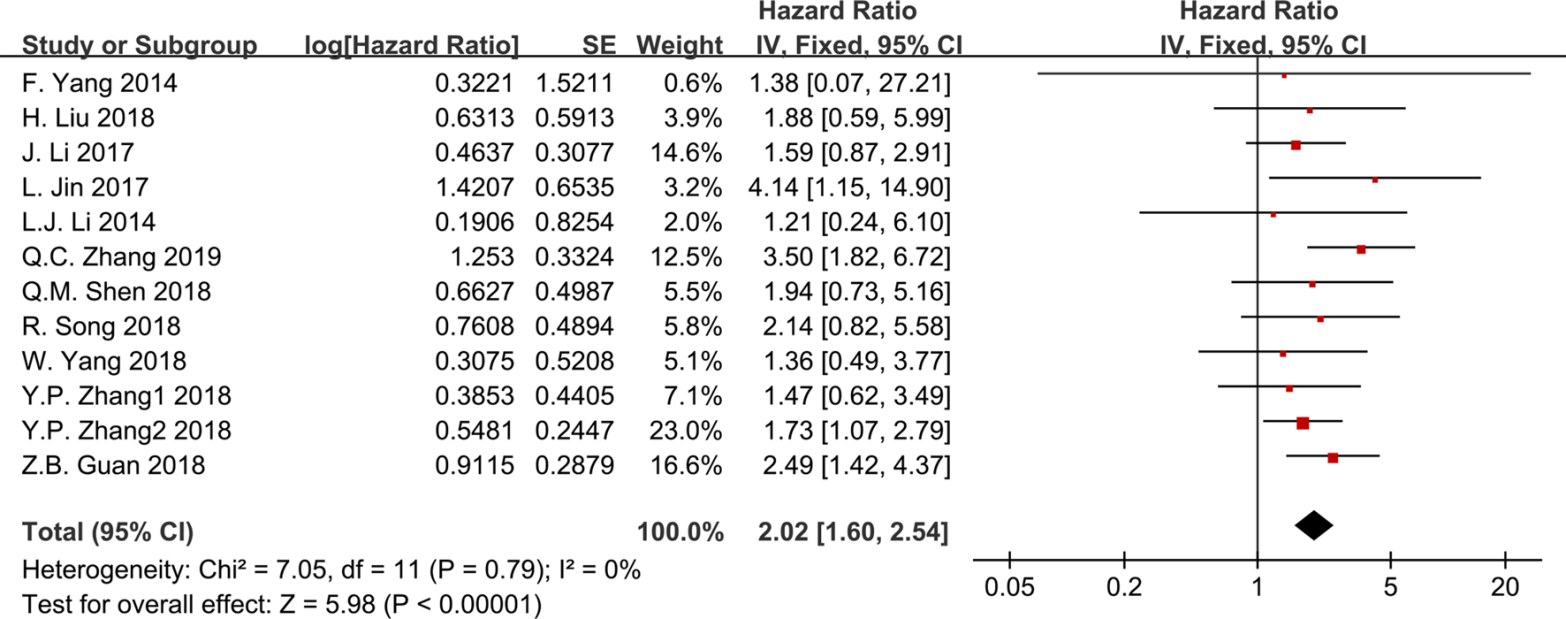
Meta-analysis for the association between GHET1 expression and OS

Twelve studies reported OS for eight types of cancer based on GHET1 expression in 1114 patients. As shown in Fig. 2, a fixed-effects model was adopted since there was no statistical heterogeneity (I^2^ = 0.0%, P = 0.783). The pooled HR for the high GHET1 expression group versus the low group was 2.037 (95% CI 1.626–2.551, P < 0.001). This pooled result indicates a significant association between overexpressed GHET1 and poor OS.

In order to further explore the potential prognostic value of GHET1, a series of subgroup analyses were performed based on cancer type, sample size, cut-off value, treatment, and NOS score. The results indicated that, regardless of the cancer type, sample size, cut-off value, treatment, and NOS score, the up-regulation of GHET1 was significantly correlated with poor OS in all subgroup analyses (Table 2).

**Table 2:** Subgroup analysis for the association between GHET1 expression and OS

Subgroups	No. of studies	No. of patients	Pooled HR (95% CI)	PHet	I^2^ (%)	P value
Cancer type						
Digestive system	6	663	1.751 [1.274, 2.408]	0.844	0.0	0.001
Respiratory system	2	157	2.363 [1.515, 3.685]	0.656	0.0	< 0.001
Others	4	294	2.377 [1.505, 3.754]	0.357	7.2	< 0.001
Cut-off value						
Median	10	867	2.067 [1.615, 2.646]	0.802	0.0	< 0.001
Others	2	247	1.894 [1.096, 3.274]	0.185	43.0	0.022
Treatments						
Surgery	10	915	1.890 [1.475, 2.420]	0.899	0.0	< 0.001
Others	2	199	2.918 [1.698, 5.015]	0.324	0.0	< 0.001
Sample size (n)						
≤ 80	5	282	2.312 [1.574, 3.394]	0.725	0.0	< 0.001
> 80	7	832	1.906 [1.444, 2.517]	0.609	0.0	< 0.001
NOS score						
≤ 7	6	449	2.456 [1.602, 3.765]	0.524	0.0	< 0.001
> 7	6	665	1.895 [1.454, 2.470]	0.850	0.0	< 0.001

*HR* hazard ratio, *CI* confidence interval

**Fig. 3:**
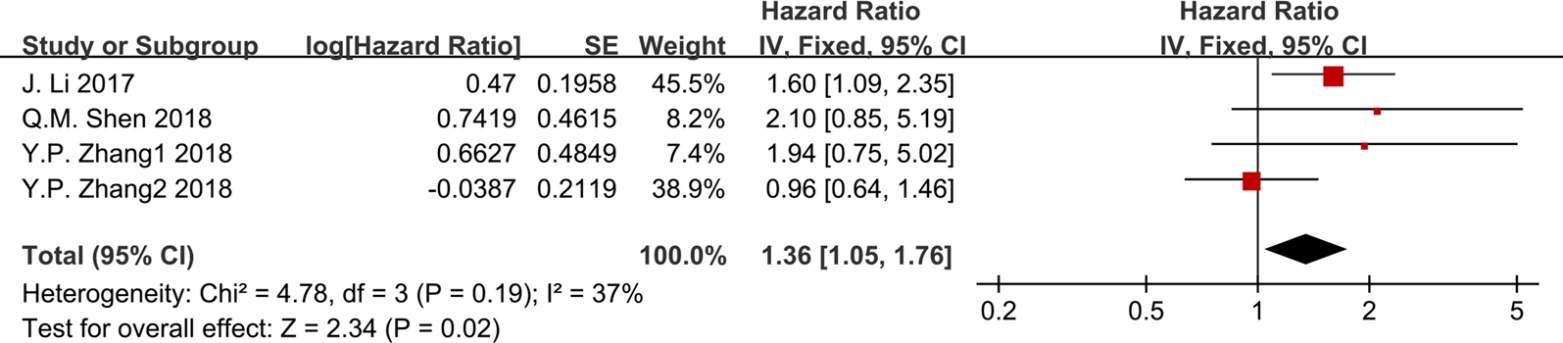
Meta-analysis for the association between GHET1 expression and DFS

Moreover, DFS was reported in four studies with a total of 572 patients. There was no statistical heterogeneity, and a fixed-effect model was utilized (I^2^ = 37.1%, P = 0.190). The pooled results indicated that elevated GHET1 expression was significantly correlated with shorter DFS (HR = 1.362, 95% = 1.051–1.765, P = 0.020, Fig. 3). Overall, these results indicate that GHET1 might be an independent factor associated with survival of cancer patients.

### Associations between GHET1 expression and clinicopathological parameters

To explore the associations between GHET1 expression and clinicopathological features, further meta-analysis of seven studies was conducted. The pooled results are shown in Table 3. Compared to low GHET1 expression, high GHET1 expression level was statistically correlated with larger tumor size (P < 0.001, fixed model), positive lymph node metastasis (P < 0.001, fixed model), positive distant metastasis (P < 0.001, fixed model), and advanced clinical stage (P < 0.001, fixed model). However, there were no statistically significant relationships between GHET1 expression level and age (P = 0.452, fixed model), gender (P = 0.925, fixed model), and histological differentiation (P = 0.467, random model). These findings demonstrate statistically significant associations between up-regulation of GHET1 and advanced clinicopathological features of cancer.

**Table 3:** Meta-analysis results for the association between GHET1 expression and clinicopathological characteristics

Variables	Studies (n)	Patient (n)	Pooled OR (95% CI)	PHet	I^2^ (%)	P	Model
Age (old: young)	15	1235	0.915 [0.725, 1.154]	0.608	0.0	0.452	Fixed
Gender (male: female)	13	1081	0.987 [0.750, 1.299]	0.828	0.0	0.925	Fixed
Tumor size (large: small)	13	1143	2.794 [2.197, 3.554]	0.101	35.2	< 0.001	Fixed
Differentiation (poor: well)	12	1010	1.266 [0.671, 2.388]	0.000	77.4	0.467	Random
Lymph node metastasis (yes: no)	9	596	4.018 [2.816, 5.734]	0.151	33.4	< 0.001	Fixed
Distant metastasis (yes: no)	6	342	3.994 [2.028, 7.864]	0.296	18.1	< 0.001	Fixed
TNM stage (III/IV: I/II)	13	1125	3.721 [2.889, 4.793]	0.336	10.9	< 0.001	Fixed

*Random* random-effects model, *Fixed* fixed-effects model, *OR* odds ratio, *CI* confidence interval

### Publication bias and sensitivity analysis

**Fig. 4:**
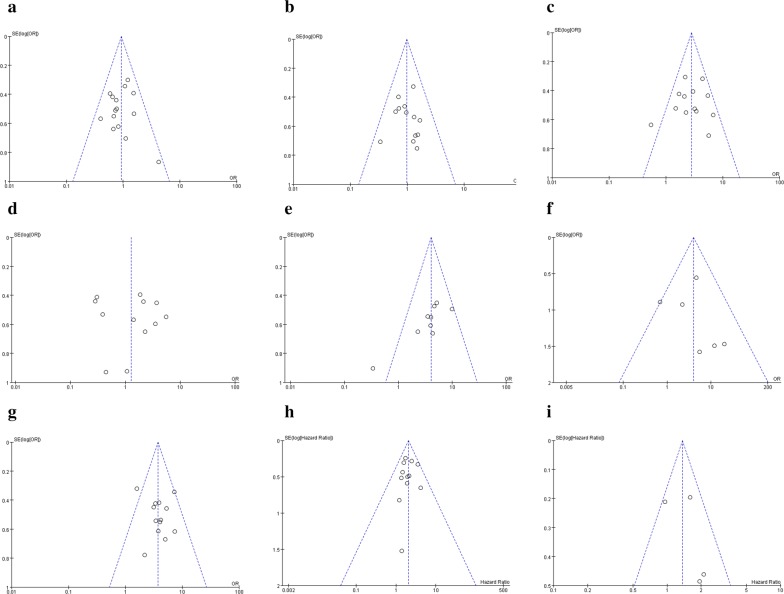
Funnel plots for the meta-analyses of the association between GHET1 expression and clinicopathological parameters or prognosis; **a**, age; **b**, gender; **c**, tumor size; **d**, differentiation; **e**, lymph node metastasis; **f**, distant metastasis; **g**, clinical stage; **h**, OS; **i**, DFS

The Begg’s test was conducted to evaluate publication bias among the studies. As shown in Fig. 4a–i, regardless of age (Pr > |z| = 0.692), gender (Pr > |z| = 0.583), tumor size (Pr > |z| = 0.583), lymph node metastasis (Pr > |z| = 0.076), distant metastasis (Pr > |z| = 0.452), TNM stage (Pr > |z| = 0.760), histological differentiation (Pr > |z| = 0.732), DFS (Pr > |z| = 0.734), and OS (Pr > |z| = 1.000), the funnel plots of the Begg’s test showed no obvious asymmetry. This indicates publication bias did not affect the pooled results in the meta-analysis.

**Fig. 5:**
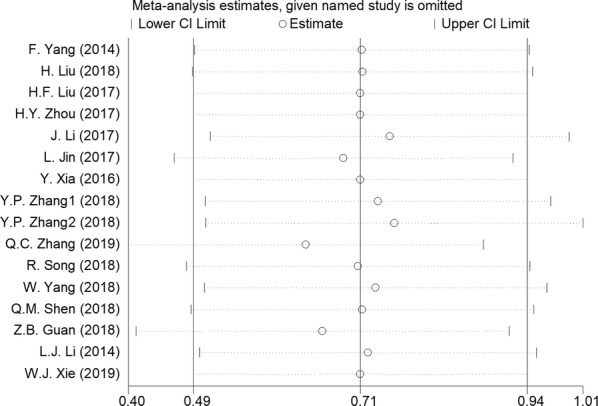
Sensitivity analysis for the meta-analysis of the association between lncRNA DANCR expression and OS

In addition, to assess the stability of the combined results, we performed a sensitivity analysis of OS. The sensitivity analysis indicated that no individual study changed the combined results; thus, the OS results can be considered reliable (Fig. 5).

### Validation of GHET1 in TCGA dataset

**Fig. 6:**
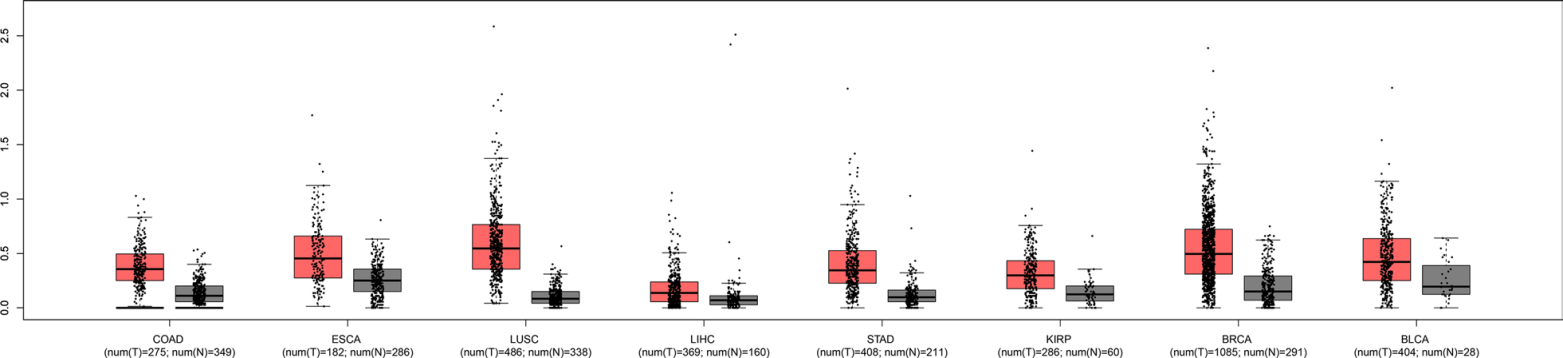
The expression levels of GHET1 in eight types of cancer tissues and normal tissues in TCGA cohort

**Fig. 7:**
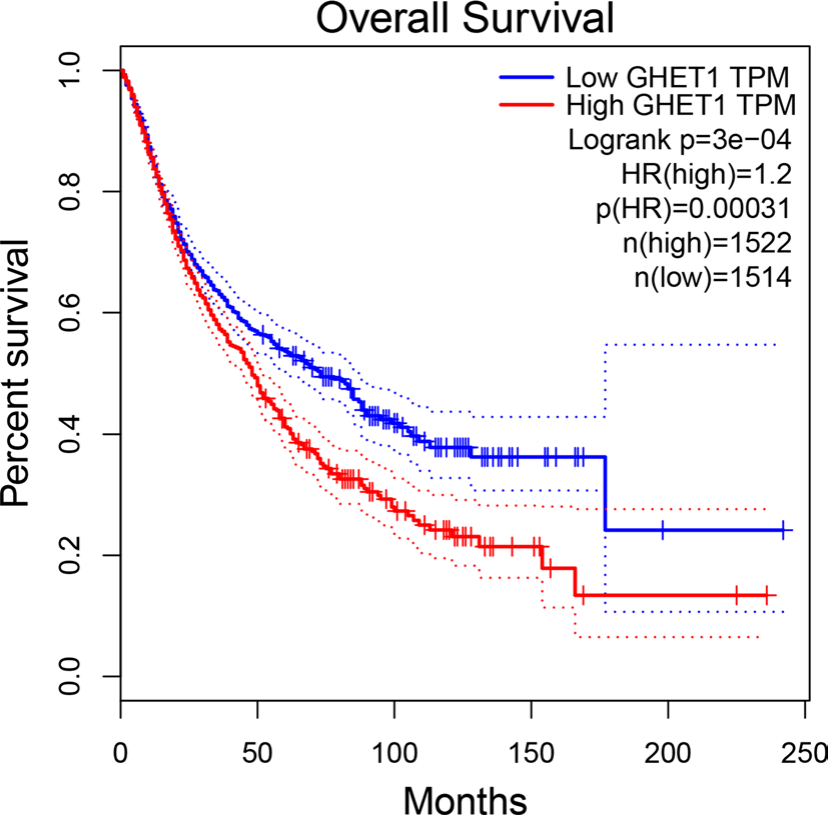
Overall survival plots of GHET1 in TCGA cohort, including BLCA, COAD, ESCA, KICH, KIRP, LIHC, LUAD, LUSC, PAAD and STAD (n = 3077, Log-rank P < 0.001)

To further verify our results, the expression of GHET1 in eight types of cancer was evaluated using RNAseqV2 and TCGA clinical data. The results indicated that GHET1 was up-regulated in most cancers, including lung squamous cell carcinoma (LUSC), kidney renal papillary cell carcinoma (KIRP), liver hepatocellular carcinoma (LIHC), stomach adenocarcinoma (STAD), colon adenocarcinoma (COAD), breast invasive carcinoma (BRCA), bladder urothelial carcinoma (BLCA), and esophageal carcinoma (ESCA) (|Log2FC| Cutoff > 1, q-value < 0.01, Fig. 6). Further, the GHET1 high expression indicated poor prognosis in several cancer (Additional file 1: Fig. S1). We merged the expression and prognosis data for cancers of the digestive system, respiratory system, and urinary system, including BLCA, COAD, ESCA, STAD, LIHC, pancreatic adenocarcinoma (PAAD), lung adenocarcinoma (LUAD), LUSC, kidney chromophobe (KICH), and KIRP. According to the median GHET1 expression level, 3036 patients with 10 cancer types were classified into two groups. As demonstrated in Fig. 7, the GHET1 high expression group had shorter OS than the GHET1 low expression group, confirming that over-expression of GHET1 is correlated with poor OS in various human cancers (P < 0.001).

## Discussion

Accumulating evidence has demonstrated that lncRNAs act as crucial regulators of almost all aspects of physiological and pathological processes [26–30]. Multiple studies have also indicated that lncRNAs contribute to the carcinogenesis and progression of several tumors [6–10]. Recently, several studies have reported that lncRNA GHET1 might be related to prognosis in cancer patients [14–24, 31]. Therefore, we performed this meta-analysis of 16 eligible studies to systematically evaluate the prognostic value of GHET1 in all cancers.

In the present study, we evaluated the prognostic value of GHET1 in cancer. The pooled HR indicated that GHET1 overexpression was significantly associated with poor OS and DFS in cancer. Moreover, further subgroup analyses indicated that elevated GHET1 expression was significantly correlated with OS in each subgroup, regardless of the analysis model, sample size, cut-off value, treatment, cancer type, and NOS score. The pooled data illustrated that GHET1 overexpression was significantly associated with larger tumor size, positive lymph node metastasis, positive distant metastasis, and advanced TNM stage. Unexpectedly, we failed to identify an association between GHET1 expression and histological differentiation. However, these results might be not reliable because there was significant heterogeneity among the included studies. Overall, high GHET1 expression was an unfavorable risk factor for survival outcomes in patients with cancer; thus, GHET1 might be a valuable biomarker for a variety of cancers. To our knowledge, this research is the first meta-analysis focusing on the prognostic value of GHET1 in human cancers.

Many studies have tried to illustrate the correlation between high GHET1 expression and cancer prognosis; however, the molecular mechanism of GHET1 remained unclear [14, 17–19, 23, 24]. Feng et al. found that GHET1 overexpression promotes gastric cancer cell proliferation by binding to IGF2BP1 and enhancing the stability of c-Myc mRNA [14]. Further, GHET1 overexpression could prohibit cellular apoptosis by promoting the expression of Bcl-2 and could contribute to the development of multidrug resistance by promoting the expression of MDR1 and MRP1 in gastric cancer [16]. Xia et al. study revealed that down-regulation of GHET1 could prohibit the G1-S phase transition of the cell cycle in gastric cancer cells by modulating the expression of P21, cyclin, and CDK [21]. Moreover, upregulation of GHET1 could be induced by hypoxia in gastric cancer cells, and the depletion of GHET1 c significantly enhanced the CpG island methylation of EGFR, which plays a crucial role in the metastasis of cancers [32]. In hepatocellular carcinoma, Ding et al. found that high GHET1 expression could be activated by H3K27 acetylation, and could promote the progression and migration of cancer by physically binding to ATF1 [20]. In addition, Jin et al. showed that GHET1 can bind to the enhancer of EZH2 and recruit PRC2 to the promoter region of KLF2; KLF2 acts as a tumor suppressor in hepatocellular carcinoma and is epigenetically repressed [24]. As for lung cancer, Guan et al. revealed that GHET1 depletion inhibited the proliferation, invasion, and epithelial-mesenchymal transition (EMT) of cancer cells by inhibiting the LATS1/YAP pathway [19]. Additionally, several studies have reported that GHET1 can promote EMT in esophageal squamous cell carcinoma, breast cancer, colorectal cancer, osteosarcoma, renal cell carcinoma, and bladder cancer [17, 18, 22, 31, 33, 34]. Other studies have also shown that GHET1 promotes the progression of cancer and might be a therapeutic target of cancer [35, 36].

The present meta-analysis has several limitations. First, this meta-analysis included only 16 studies, and all of these studies were from China. Therefore, the results might only apply to Asian or Chinese patients, which may limit the representativeness of the results. The validation tests using data from TCGA make up this disadvantage in some extent. Second, the HRs and 95% CIs in several studies could not be directly obtained. Thus, we extracted the data from the Kaplan–Meier curve in these studies, which might introduce statistical errors. Finally, the sample sizes of some cancer types in this meta-analysis were limited; this may have contributed to the heterogeneity and may have affected the reliability of the pooled results for some cancer types. Nevertheless, the results of this meta-analysis should be verified by studies evaluating more cancer types with larger sample sizes.

## Conclusions

In sum up, the up-regulation of lncRNA GHET1 expression was significantly associated with poor OS, poor DFS, and advanced clinicopathological characteristics in various cancers. GHET1 can be considered to be a promising prognostic predictor for human cancers. However, high-quality studies with larger samples sizes and those encompassing more cancer types are still needed to verify these conclusions.

## Supplementary information

**Additional file 1: Fig. S1.** OS plots for each TCGA cohort.

## Data Availability

The datasets during and/or analysis during the current study available from the corresponding author on reasonable request.

## Abbreviations

BC: Breast cancer
BLC: Bladder cancer
ESCC: Esophageal squamous cell carcinoma
GC: Gastric cancer
HCC: Hepatocellular carcinoma
PADC: Pancreatic cancer
NSCLC: Non-small lung cancer
qRT-PCR: Quantitative reverse transcription polymerase chain reaction
CP: Clinicopathological parameters
OS: Overall survival
DFS: Disease-free survival
HR: Hazard ratios
OR: Odds ratio
CI: Confidence intervals
NOS: Newcastle–Ottawa Scale
