# Supplementary material for: Prognostic value of long non-coding RNA GHET1 in cancers: a systematic review and meta-analysis
Source: Cancer Cell Int. 2020 Apr 7;20:109. doi: 10.1186/s12935-020-01189-9 (PMC7137500; doi:10.1186/s12935-020-01189-9)
Supplement: Supplementary file 1 — Additional file 1: Fig. S1. OS plots for each TCGA cohort. [file 12935_2020_1189_MOESM1_ESM.docx]

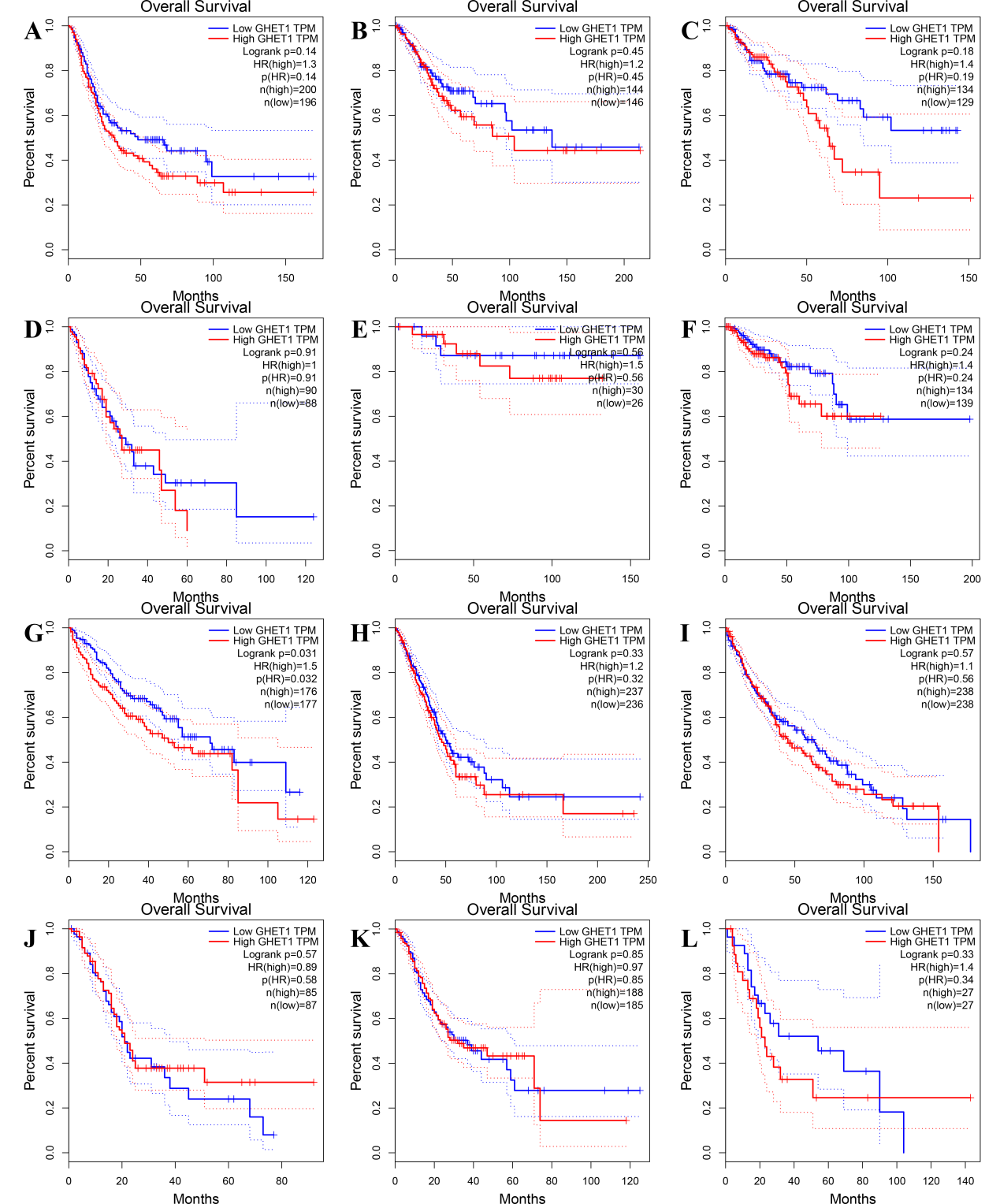


Supplementary Fig1. OS plots for BLCA(A), CESC(B), COAD(C), ESCA(D), KICH(E), KIRP(F), LIHC(G), LUAD(H), LUSC(I), PAAD(J), STAD(K), UCS(L)
